# News and Miscellany

**Published:** 1882-10

**Authors:** 


### NEWS AND MISCELLANY

Noah Cressy, M.D., V.S., Ph.D., has taken the editorship of the Hygienic and Veterinary Department of the *National Live Stock Journal.*

Feeding Horses.—It is not good policy to let work horses get thin. It costs more to put on flesh than it does to keep it on. Flesh that becomes hardened by exercise will be kept up with less food, under the same work, than it took to put it on. From fifteen to thirty pounds of food will about supply the daily consumption of horses, large and small.

The English cavalry horses are fed ten quarts of oats and twelve pounds of hay three times a day. The American cavalry horses have had the English rations increased to thirteen or fourteen quarts of oats and an equal amount of hay three times a day. The hunter, in the season, is allowed from sixteen to eighteen quarts of oats and about eight pounds of hay, fed five times a day. The race horse is allowed from eighteen to twenty quarts of oats per day, and nearly as much hay as the hunter, being usually fed five times a day.—*National Live Stock Journal.*

The Pulse of the different domestic animals :

| Species. | Mean Number of Pulsations per Minute. | | |
|---|---|---|---|
| | Adultism. | Youth. | Old Age. |
| Horse, . . . | From 36 to 40 | From 60 to 70 | From 32 to 38 |
| Ass and Mule, . . | " 46 to 50 | " 65 to 70 | " 55 to 60 |
| Ox, . , . . | " 45 to 50 | " 60 to 70 | " 40 to 50 |
| Sheep and Goat, . . | " 70 to 80 | " 85 to 95 | " 55 to 60 |
| Pig, . . . . | " 70 to 80 | " 100 to 110 | " 55 to 60 |
| Dog, . . . . | " 90 to 100 | " 110 to 120 | " 60 to 70 |
| Cat, . . . . | " 120 to 140 | " 120 to 140 | " 100 to 120 |

—*National Live Stock Journal.*

The cow population of the United States is 12,611,148, or about one cow to every four people. This only includes milch cows, and their value is estimated at $340,500,996, an average of $27 per head, based upon their prices in different States. There are comparatively but few cows in the South,

Louisiana, for instance, reporting but 115,000, while Iowa, with about the same population, reports 845,000. The principal dairy States report as follows:

| | Number. | Value. |
|---|---|---|
| New York, | 1,474,700 | $46,349,821 |
| Pennsylvania, | 844,800 | 27,248,025 |
| Ohio, | 693,000 | 21,739,410 |
| Michigan, | 421,100 | 12,763,541 |
| Indiana, | 439,100 | 11,710,797 |
| Illinois, | 716,400 | 19,701,000 |
| Wisconsin, | 448,700 | 12,388,607 |
| Minnesota, | 344,600 | 8,432,362 |
| Iowa, | 845,100 | 22,237,700 |
| Missouri, | 547,700 | 10,294,000 |
| Kansas, | 409,800 | 10,098.876 |
| Nebraska. | 188,600 | 5,594,762 |
| California, | 487,600 | 16,051,792 |

Natural History and "Education."—The following story is narrated by a correspondent in the *Students' Journal:* An English physician had recommended a patient to take plenty of horse exercise, so the gentleman went to a dealer's and looked at a horse, with the intention of purchasing it. He came back to the doctor with the following report: "I went," he said, "to the horse-dealer's and saw a horse; he looked a very nice, steady animal, and I was going to buy him, but remembering it was a proper thing to look into a horse's mouth—but why I could not recollect—I put on my spectacles and examined his mouth. I was then delighted that I had not completed the purchase, for I found that the poor suffering creature had lost nearly all his teeth; the only ones left that I could see being six on the top and six in the bottom jaw; and then came two great gaps without any teeth at all. I told the horse-dealer it was cruel to keep a poor animal like that without any teeth. The dealer assured me the animal had several more teeth in his jaws farther back, and I might put my hand in and try if I liked. I indignantly refused, however, to be taken in in this manner." Evidently in some "educated circles" the advance in natural history is quite astonishing.

Veterinary Honors.—It gives us much pleasure to learn that our distinguished colleague, M. Zundel, of Strasbourg, was in June last elected a corresponding member of the Belgian Academy of Medicine. Professor Bouley, inspector of the French Veterinary Schools, has been elected president of the Acclimatization Society of France. The Paris Academy of Medicine has awarded the Barbier prize of 6,000 francs (£250) to Professor Toussaint, for his researches in anthrax, fowl cholera, and experimental acute septicæmia. At the graduation ceremony of the Edinburgh University, held on August 1st, Mr. J. McFadyean, professor of anatomy in the Dick Veterinary College, received the degree of Bachelor of Medicine and Master in Surgery. At the distribution of prizes and conferring of degrees in the Toronto University on June 8th, in the Faculty of Medicine, the University silver medal was bestowed on J. T. Duncan, Professor of the Ontario Veterinary College.—*Veterinary Journal.*

The record of casualties in the conveyance of live stock by sea, taken from the returns furnished to the Privy Council by the Inspectors of the ports where American and Canadian cattle are landed, presents a doleful picture of the sufferings of these unfortunate dumb passengers. It appears that in last year alone 8,721 animals were thrown overboard, and 498 were landed dead, while 472 were so much injured and exhausted that they were killed at the place of landing. This makes a total of 9,221 animals which, in the space of twelve months, were either lost or seriously injured in the passage across the Atlantic.—*Drovers' Jonrnal.*

Veterinary Department at Harvard.—On the 28th day of September a veterinary department will be opened in Harvard University, and the regular course of the veterinary students will be three years. The subsidiary branches to be studied by the veterinary aspirant are taught in the other departments of the university, while ample preparations will be made for the theoretical and practical instruction in the veterinary art. The addition of this new department will be specially welcome to the many patrons of veterinary surgery, and will give much satisfaction also to the friends of the university, who will watch with pleasure its constant progress.

Unknown Disease Among Cattle.—The failure of Congress to pass a measure to prevent the spread of contagious diseases among cattle is likely to be brought prominently into notice by the ravages of a strange disease which has recently made its appearance among stock in several States. In Pennsylvania, in the neighborhood of Reading, the disease appeared, and spread with alarming rapidity in two townships, in spite of a rigid quarantine. The Agricultural Department received information during the past few weeks of the breaking out of a similar plague among the cattle in the States of Alabama, North Carolina, Virginia, and near Wheeling, West Virginia. It is believed at the Department that the disease is what is known as Texas fever, or splenic fever, the stock dying from apoplexy of the spleen. Dr. Salmon, one of the Department inspectors, who has been in Virginia, near Abingdon, where the disease appeared, has been sent to West Virginia, declares it to be a virulent form of splenic fever. Mr. LeFevre, of Ohio, who has labored in two Congresses to secure the passage of the Pleuro-pneumonia and Contagious Diseases Bill, said that had the bill become a law, its value would be shown now, as it provided for a commission of experts whose duty it would have been to go wherever the disease appeared and take summary measures for its suppression or confinement within limits. The House passed the bill four months ago, but it failed to pass in the Senate.

The bill referred to above, which failed to pass the Senate, provided for the creation of a bureau whose chief shall be a competent veterinary surgeon, and whose duty it shall be to investigate and report upon the number, value. and condition of the domestic animals of the United States, their protection and use, and also inquire into and report the causes of contagious and communicable diseases among them, and to collect such information on these subjects as shall be valuable to the agricultural and commercial interests of the country. The Commissioner of Agriculture is also authorized to employ two commissioners, one of whom shall be a practical stock-raiser and one an experienced business man, familiar with questions pertaining to commercial transactions in live stock, whose duty it shall be to advise with regard to the best methods of treating, transporting, and caring for animals, and of providing against the spread of said diseases. The compensation of the commissioners is to be ten dollars a day, with all necessary travelling expenses, while engaged in the performance of their duty. The salary of the chief of the bureau is to be three thousand dollars per annum; and the Commissioner of Agriculture is to appoint a clerk for the bureau, with a salary of eighteen hundred dollars per annum. The Commissioner of Agriculture is directed to make inquiry through the chief of the Bureau of Animal Industry as to the existence of pleuro-pneumonia or any contagious or communicable disease along the dividing line between the United States and foreign countries, and along the lines of transportation from all parts of the United States to ports from which live stock are exported, and to make a report of the results of such investigation to the Secretary of the Treasury. He shall establish such regulations concerning the exportation and transportation of live stock as the results of the investigation may require.

The Cattle Quarantine.—The Canadian quarantine of Quebec has been visited and its practical workings carefully studied by the commission. Sites have been selected for quarantines at Portland, Boston, New York, Philadelphia and Baltimore, subject to the approval of the Secretary of the Treasury, and accommodations for importers at these points will speedily be provided at government expense. The commission will,as soon as possible, prepare regulations for the government of these quarantine stations, and the Secretary of the Treasury and the collectors of the ports abovementioned will be relieved from what has been for the past two years a constant source of annoyance to them on account of the attempt to enforce a quarantine without any provisions for the preparation of suitable quarters for the animals to be quarantined.—*Breeders' Gazette.*

A Horse with a Grievance.—The history of a cart-horse foaled in 1880 may be said to constitute a grievance that should be somehow redressed. It is not easy for a breeder to rear the best year-old agricultural stallion, and when he does so, it is very hard for his exhibit to be disqualified from carrying off prizes unless the cause of disqualification is, beyond all controversy, palpable and decided. The case to which I would draw attention is the first prize agricultural stallion, foaled in 1880, which in class 3 at the late Royal Show at Reading was awarded chief honors. It is named Certainty, and is the property of the Hon. E. K. Coke, who should know something of a good horse. Now, the history of this horse demands that the conditions at all first-class shows should be established on a general standard of excellence and soundness, and it may be submitted that, in special cases, final judgment as to soundness should be reserved. Doubtless any power of appeal would be attended with inconvenience; but even inconvenience had better be suffered than injustice.

Now, in the matter of the exhibition of the above horse at the Cart Horse Show (where it was entered under the name of Reality), the breeder had sold him to the exhibitor for a certain sum, but in the pride and confidence of his heart in the goodness and soundness of the animal, he had stipulated that he, the breeder, was to receive the value of any prizes which the colt (that had already won several) might gain at the Cart Horse Show. This was a part of the sale bargain. Professor Pritchard a short time previously to exhibition has passed Reality as sound, and when he appeared at Islington the Lincolnshire horse won the undisputed good opinion of the judges, and also it may be said of the critical public. However, on being submitted to veterinary inspection, Reality's pride of place was lost, the animal being pronounced unsound from spavin. Here was a great disappointment and surprise to breeder and exhibitor; but there was no appeal from the verdict. So little weight, however, had this decision at the auction, that bids ran up to £250, at which high figure for a colt he was bought in, a fact showing the strong confidence of the owner.

Subsequently, a few days later, the Hon. Mr. Coke (after having had the animal examined by an eminent veterinary surgeon, who found him sound) bought him and to get rid of the ill-luck that had attached to "Reality" re-christened him "Certainty," entered him for exhibition at the Royal with the results already mentioned. In fact, admiration has followed this young cart stallion wherever he has been exhibited, but the breeder has not got rid of his grievance, nor can the public consent to a horse being pronounced unsound from spavin at one show, and being acknowledged sound shortly after by the Royal authorities. Is spavin a disease to be cured between the dates of the Cart Horse Show and Reading ? Would not an animal unsound at one be unsound at the other show ? Can veterinary doctors disagree over a case of spavin ? Is the old common belief of farriers and horse dealers that spavin cannot be cured without leaving blemish and without taking up a long time, to be given up ? or is it not possible that in this case, had there been a reserve appeal, some error of judgment would have been revealed, and the stigma of unsoundness removed ? A bad name to a dog hangs him; and a verdict of unsoundness sticks to a horse, and, if untrue, does injustice to breeder and owner Young horses often have enlarged hocks, which fine down as they grow older; and the large cushion-bones of coarse hocks are scarcely a disadvantage to horses of the cart breed. Captain Hayes, in his "Notes for Horse Owners," says, quoting the high authority of Percivall, "Nothing is more common than to meet with horses, colts even, who have what the dealers call 'knots' in their spavin places; and the time was when such 'knots,' which have always been regarded as spavins, were certificated as constituting unsoundness. This was professional decision which met with a good deal of opposition at the time, and justly so, and the result has been that such 'knots' are now allowed to pass as compatible with soundness." In bone spavin there is a deposition of bone, as the result of inflammation, on the inner and lower part of the hock, and its position high or low down makes the spavin either serious or almost unimportant. In occult spavin no external evidence of disease can be observed, although lameness of an inveterate form may result from the ulceration of the surfaces of the bones which form the joints. Whether bone or occult spavin, it is inflammation that constitutes the disease, and by the cessation of inflammation the reparative process begins. Hereditary predisposition is well marked in spavin, and thus to ticket a stallion with spavin-unsoundness greatly reduces his value for breeding purposes. Major General Sir F. Fitzwygram, Bart. (in his work "Horses and Stables") has a lucid and valuable chapter on spavin and its treatment, writes squarely, "Spavins cannot be removed when once fully formed; absorption of the deposit may, to a certain degree, be assisted by the application of mild blisters, or iodine, or setons, but, when consolidated, the bony deposit will not yield to any treatment." That errors of judgement may occur, the following passage shows: "No enlargement can safely be said to be spavin, until, by manipulations, it has been ascertained to be bone. Without such manipulation, other enlargements, such as a distended vein, or a thickening of integuments, resulting from a blow, may be mistaken for spavin."

In conclusion, it seems to me that Certainty is a "horse with a grievance," and breeders, exhibitors and the public should know the reason why he was unsound at Islington, and sound at Reading.—*London Field.*

Bovine Post-Mortem.—From the *British Medical Journal* we learn that it has become customary in Bengal for cattle to be poisoned on account of real or imaginary affronts. At the suggestion of the surgeon general all surgeons hereafter will superintend the evisceration of the animal, a butcher doing the work. Tableaux—big umbrella, surgeon under it, coolies to fan him, butcher at work over a half or wholly putrid cow, thermometer 120 degrees in the shade. Truly, the life of a medical man in Lower Bengal has not fallen upon pleasant places.

Ought Diseased Meat to be Used as Human Food?—A medical officer of health writes the following to the *Lancet*: "I wish, through the medium of your columns, to ask the opinion of medical officers of health on the following case:

"A cow, which had been previously ailing, gives birth to a calf, and on the following day it is slaughtered, the meat dressed and brought into market. I am consulted as to the flesh being fit for human consumption, and finding on examination that it is soft and flabby and dark in color, with an alkaline reaction to litmus paper, and taking into consideration that the cow had been under veterinary treatment for several days, had been killed so soon after parturition, and had had physic administered to it, I condemned the meat. Acting on my advice, the Board of Health instituted an action against the butcher, and the case is heard before the local magistrates, who, after a lengthy trial, find the meat not unfit for human food.

"Was I justified in condemning the meat? The magistrates by their verdict say I was not, but, as this may be made a test case, I should like the opinion of any of my medical brethren who may feel interested in the matter, and who may feel qualified to speak on the subject. I should perhaps mention that a practitioner from a neighboring town gave evidence on the opposite side, and gave it strongly as his opinion that such meat should not be condemned. He had not seen the meat. I should like to know, seeing that all authorities on the subject were entirely disregarded, what are the circumstances under which meat ought to be condemned and destroyed as unfit for human food."

[It is impossible from the data above given for us to say whether the meat was fit for food. We may say, however, that it is not the best way for inspectors to say that meat is either fit or entirely unfit for consumption. The plan recommended by German and other experience is to divide the meat into different grades, either three or four, the first being perfectly good, the last perfectly bad.—Ed.]

Arab Horses.—Of the eleven Arab horses comprising the Crabbet Stud, recently sold, six only appear to have been purchased to remain in England; two of them go to the Colonies, two to the Continent, and one, the entire horse Pharaoh, to Hungary, his price being £525. It is to be regretted that so fine an animal, with his rare pedigree, was not retained in England. The stamina of the English racehorse is open to some reproach, seeing that there are so few that can at full speed compass a distance much exceeding two miles. If the same rule holds good with the English thoroughbred as it does with sheep—which is, that it is necessary at certain intervals of time to take a dip into the blood of the pure, unmixed, original breed, in order that the main character of the animal shall be maintained in some specific shape or other—then should a fresh infusion of the blood of the Arab, with his undoubted stamina, be again blended with our English racehorse.

No animal existing has a history so interesting as the horse, whether in ancient or modern times. The books of Genesis, Exodus, Kings, and Job contain passages descriptive and poetical, showing that he had been long under the control of man. Solomon, who had a stud of 40,000 horses, used to recruit his numbers from Egypt, and the price is given as 150 shekels, or £20. The East, no doubt, can claim the horse as its original home, and although he differs in form and size in different countries, it is from the influences of climate and cultivation, running through thousands of years, that the drayhorse, the Shetland pony, and the racehorse all had one common origin. Friezes of ancient temples, carving on Egyptian monuments, and Greek sculptures in the British Museum all depict the horse as we now see him in the Arab. From the days of Solomon to Mahomet, on to the Mamelukes, who were created from slaves in the twelfth century, and made into cavalry to resist the inroads of Genghu Khan and the deeds of Saladin, the Arab horse was a great factor in all these gigantic expeditions. The Mamelukes, who protected Egypt and Arabia for six hundred years, met with a great disaster from Napoleon in 1789 under the pyramids; and in 1811 they were finally crushed out by Mehemet Ali.

A superior class of Barb horses were rescued from the remains of the Spanish Armada, with which the heavy English-bred mixed with great success, as in James's reign horse racing assumed a more prominent feature at Croydon and Newmarket. James gave silver bells as a trophy to the winners; but Charles II. introduced silver cups in the place of bells. It was in Charles's time (1684) at the siege of Vienna by the Turks, that an interesting episode in the history of the Arab horse took place. A Turkish bashan and suite were captured, and three of their steeds were brought to England, on speculation, for sale to his Majesty. One of these horses is thus described: "A bright bay, two white feet, a blaze; such a head, eyes, ears, neck, breast, belly, haunches, legs, pasterns, and feet—in all regards beautiful, and proportioned to admiration; spirited, proud, nimble, making halt, turning with swiftness in so small a compass that was admirable to behold. With all this so gentle and tractable as called to mind a reproach to our grooms, who bring up their horses to retain their ill habits." They were shod in iron, the hoof covered, a hole being left open at the bottom. Charles and the Duke of York were delighted, and 500gs., 300gs., and 200gs. were the prices asked for them—a price, according to the value of money then and now, equal to that paid for our best racers. Some years after the introduction of these superb Arabs came the Darley and the Godolphin Arabians, and the English-bred Childers came upon the scene in 1715. What *he* did, how that he ran over four miles at a greater speed than our boasted flyers in the present day can cover five furlongs, is matter of history, and is fully chronicled in the deeds of the turf. Eclipse died in 1789; his heart weighed fourteen pounds. Have we had anything in horseflesh at all to be compared to these two half-bred Arabs? It is a hundred years since these deeds were done, and if they are to be attempted again new blood must be infused into our stock.

It is an error to suppose, as many do, that there is but *one* breed or class of Arab horse. There are two distinct breeds—one is the "Kadesha," or of unknown descent, and which does the drudgery of the country; the other is the "Kohlani"—that is, of ancient and noble pedigree, and used for riding only. It is of this noble pedigree that the Arab is so jealous, and very rarely can an animal be obtained from the sheiks. Europe owes little to the Arabs, but there are two things certainly we are indebted to them for—one is our alphabet, and the other is the art of keeping a pedigree of a faithful and beautiful animal.—*Land and Water.*

The census of live stock taken in 1880 shows that on the farms of the United States there were at that time 10,357,981 horses, 1,812,932 mules and asses, 993,970 oxen, 12,443,593 milch cows, 22,488,500 other cattle. 35,191,656 sheep, and 47,683,951 swine. The increase in percentage over the preceding census was: Swine, 90 per cent.; cattle, 66 per cent.; cows, 39 per cent.; mules and asses, 61 per cent.; horses, 45 per cent.; sheep, 24 per cent.; while oxen showed a decrease of 25 per cent. This census is particularly interesting to the veterinarian, as it indicates the field he has to work in and the tendency of farmers in the breeding of stock. According to this showing there is one cow to every four persons, about six sheep to every four humans. while swine are so numerous that nearly every man, woman and child in the country could have one apiece if they were divided.

A Novel Cure for Hydrophobia.—A certain Dr. Sailor, Easton, Pa., had, during a long life, the reputation of preventing hydrophobia. Over thirty years ago, in my community, a boy was bitten by a dog, supposed to be rabid. He was taken to Sailor, who writing three words on a slip of paper, ordered the boy to eat it, in the meantime uttering some incantations (pow-wow) In three weeks symptoms of hydrophobia were thought to be manifesting themselves, but they passed off through the efficacy of the eaten paper! Now, unfortunately for the doctor's reputation, but fortunately for the credulity of the community, the dog had not been at once killed, neither had he died.

Trotters for New Zealand.—G. W. Griffin, the United States Consul at Auckland, New Zealand, sends a long letter to the State Department in relation to horse breeding in that country and the growing popularity of trotting there. He says: "Mr. J. J. Miller recently visited the United States, and while in Kentucky was fortunate enough to procure, among other fine horses, the celebrated trotter Contractor. Contractor was purchased for the sum of $6,000, and his safe arrival at Melbourne created much excitement. He is 15½ hands high, eight years old, and is regarded as a perfect gem. He possesses a good temper, and a good constitution, and his breeding properties are undeniable. He is an inbred Hambletonian, and is related to Dexter, George Wilkes, and St. Julien, and is said to show back to fifteen strains of the celebrated Messenger, the father of American trotters. Mr. Miller also purchased a very handsome Kentucky mare of a bright black c lor, called Tilda, by Mambrino Patchen, from Edwin Frost's mare, and sister to Rothschild, Silver Chief, and Hailstorm.

The Pacific Mail steamer City of Sydney brought to Auckland, on her last trip from San Francisco, eleven thoroughbred American trotters. These horses were purchased in Kentucky for Mr. Robert Wilkins, a gentleman residing at Christchurch, where it is intended to lay down a track. The following are the pedigrees of some of Mr. Wilkins' American trotters: Stallion Blackwood Abdallah, b. c., four years, by Homer, dam by Blackwood; bay colt, yearling, by Harold, dam by Belmont; bay filly, three years, by Messenger Chief, dam by Davy Crockett, Jr.; chestnut filly, three years, by Vanderbilt, dam by Hughes' Forest; bay filly, four years, by Garrard Chief, dam by Kolmell's Belshazzar; bay mare, five years, by Tom Stamp, dam by Bourbon Chief; brown mare, four years, by Mambrino King, dam by Flying

Cloud. The enterprise at Christchurch, like that of Victoria, is being watched with no little anxiety, and in the event of success, which very few now question, there will, of course, be other and larger importations of this kind of horses from the United States, the home of the fastest trotters in the world. Indeed, there is every reason to believe that the importation of American horses to New Zealand will prove in the near future a very profitable business. Those that arrived here have attracted no little attention. Many flattering notes have been given them in the newspaper press, and the Auckland *Herald* says that these Kentucky trotters mark a new era in the history of stock-raising in New Zealand."

